# Effect of vitamin E on energy metabolism indicators and gill tissue structure of crucian carp (*Carassius auratus*) under cooling stress

**DOI:** 10.1038/s41598-024-66327-z

**Published:** 2024-08-22

**Authors:** Jiaming Tang, Gongyan Li, Dongjie Chen, Lexia Jiang, Baosheng Huang, Peihong Jiang, Changfeng Zhang, Xiaoming Qin

**Affiliations:** 1 Shandong Key Laboratory of Storage and Transportation Technology of Agricultural Products, Shandong Institute of Commerce and Technology, Jinan, 250103 China; 2 College of Food Science and Technology, Guangdong Ocean University, Zhanjiang, 524088 China; 3 National Engineering Research Center for Agricultural Products Logistics, Jinan, 250103 China; 4 Shandong Guonong Logistics Technology Co., Ltd., Jinan, 250103 China

**Keywords:** Vitamin E, Crucian carp, Stress, Energy metabolism, Gill tissue structure, Physiology, Anatomy, Biomarkers

## Abstract

The aim of this work is to examine the effects of vitamin E addition to water on the structure of the gill tissue and energy metabolism of crucian carp (*Carassius auratus*) under cooling stress. The crucian carp were chilled using a cold acclimation intelligent chilling equipment from 20 °C to 5 °C. They were divided into three groups: the control group (E1), the negative control group (E2), and the 100 mg/L vitamin E (E3) solution. Three different temperature points (20 °C, 10 °C, and 5 °C) were used to collect, test, and analyze the samples. The findings demonstrated that in the E3 treatment group, phosphoenolpyruvate carboxykinase, acetyl coenzyme A carboxylase, total cholesterol, urea nitrogen, triglyceride, and fatty acid synthase contents were significantly lower under cooling stress than those in the E1 and E2 treatment groups (*P* < 0.05). The E3 therapy group had significantly greater blood glucose, glycogen, and glycogen synthase levels than the E1 and E2 treatment groups (*P* < 0.05). The levels of pyruvate kinase in the E1, E2, and E3 treatment groups did not differ significantly. Crucian carp's gill tissue changed under cooling stress, including capillary dilatation, and the E3 treatment group experienced less damage overall than the E1 and E2 treatment groups. In conclusion, supplementing water with vitamin E to treat crucian carp can decrease damage, improve the body's ability to withstand cold, and slow down the stress response brought on by cooling stress. This provides a theoretical basis for supplementing water with vitamin E to fish stress relief.

## Introduction

Low-temperature the primary method of anhydrous live transportation is to use low temperatures to induce dormancy in aquatic items, which are then transported in an anhydrous or misty form^1,2^. One of the key environmental elements influencing fish physiology and behavior is water temperature. Low temperatures have the potential to be fatal due to their extreme stress-inducing effects, including major tissue damage^3,4^. There are three categories of stress responses: primary, secondary, and tertiary^5^. Primary reactions occur in aquaculture animals at the level of their endocrine and neurological systems. Primary reactions can result in secondary reactions, which are modifications to different tissues and organs such the immune system, cardiovascular system, and respiratory system. Third level reactions include behavioral, growth rate, illness resistance, and reproductive changes that occur at the individual or group level as a result of secondary reactions. In order to provide the energy needed to withstand stress, the secondary stress response involves an increase in energy metabolism, which is primarily characterized by an increase in blood glucose and circulating lipids^6,7^. Studies have found that low temperature can promote the glycogenolysis of liver and muscle in turbot, increase blood sugar level, promote energy metabolism, so as to adapt to the cold environment^8^. Lipids refer to the general term of oil, fat, and lipid-like substances. Fish decompose lipids to provide energy for the body, and lipids are also an important component of cell membranes. These two biological functions are closely related to the cold tolerance of fish^9^. Therefore, it is crucial to research ways to lower fish energy metabolism in order to slow down the stress reaction during the cooling process. This will help fish become more resilient to stress and preserve the quality of aquatic products.

Vitamin E functions as an antioxidant in aquaculture, protecting fish cell membranes and preventing oxidation and stress^10^. Relevant research has demonstrated that vitamin E can improve the antioxidant capacity, boost immunity, and regulate disease resistance in a variety of fish species, including yellow croaker (*Larimichthys crocea*), mottled grouper (*Epinephelus moara*), and pearl gentian grouper (*Epinephelus lanceolatus*)^11–13^. Prior research has demonstrated that crucian carp stress can be reduced by adding vitamin E to the water body. The greatest reduction in the body stress reaction resulting from cooling stress is achieved with an aqueous solution containing 100 mg/L of vitamin E^14^. However, there is currently a lack of research on how the addition of vitamin E to the water body affects the markers of energy metabolism in fish blood of stressed fish. This study investigated how vitamin E treatment affects the changes in gill tissue structure, lipid, and carbohydrate metabolism of crucian carp during cold stress. From the perspectives of histology, lipid and carbohydrate metabolism, and energy metabolism, the study examined the effects of vitamin E on crucian carp under cold stress and the structure of their gill tissue. The findings of the study will offer theoretical references for the investigation of vitamin E addition to water bodies as a means of mitigating fish cold stress.

## Materials and methods

### Materials

After being bought from Jinan's seafood market, crucian carp (*Carassius auratus*) were shipped to the National Engineering Research Center for Agricultural Products Logistic. Adult fish measuring 0.28 ± 0.09 kg at birth, 15.73 ± 1.12 cm in length, perfect physical condition, complete scales, and consistent size were chosen and housed in an aquatic product temperature-controlled circulating water filtration system for a short period of time. Temporary parameters included a water temperature of 20 ± 1 °C, dissolved oxygen levels of ≥ 6 mg/L, and a 24-h incubation period. In the reported experiments and methods involving live vertebrates, all relevant guidelines and regulations, especially the ARRIVE Guidelines27, have been fully taken into account and observed17. Additionally, This study was approved by the Research Ethics Committee of Guangdong Ocean University, China (GDOU-LAE-2023-033).

### Instruments and equipment

BK-280 automatic biochemical analyzer, Shandong Boke Biological Industry Co., Ltd.; t9S ultraviolet spectrophotometer, Beijing Puxi General Instrument Co., Ltd.; Multiscan MK3 Microplate Reader, Semmerfeld (Shanghai) Instrument Co., Ltd.; intelligent cold acclimation/wake-up box for fresh aquatic products independently developed by the Key Laboratory of Agricultural Products Storage and Transportation Technology in Shandong Province (Patent No. ZL201310447777.8); aquaculture and circulating water treatment system, Qingdao Zhongke Seawater Treatment Equipment Engineering Co., Ltd.; and JPB-607A Portable Dissolved Oxygen Analyzer, Shanghai LeiMag Electric Scientific Instruments Co., Ltd.

### Test method

#### Vitamin E treatment

Crucian carp were treated with two vitamin E aqueous solutions at concentration gradients of 0 and 100 mg/L. One of them, the crucian carp treated with 0 mg/L vitamin E aqueous solution, was designated as the control group (E1) and consisted of 18 crucian carp. The other group, the crucian carp treated with 100 mg/L vitamin E aqueous solution, was designated E3 and consisted of 18 crucian carp. Furthermore, an E2 negative control group was also created. To simulate whether ethanol would have an impact in the experiment, the E2 treatment group is a crucian carp group treated with an ethanol aqueous solution. The ethanol content of the ethanol aqueous solution is the same as the amount of ethanol used to prepare the 100 mg/L vitamin E aqueous solution. The number of experimental fish used in this treatment group is also 18 crucian carp. All groups were repeated in triplicate. The temporarily reared crucian carp were transferred into a cold acclimation intelligent cooling device containing 0 mg/L, 100 mg/L vitamin E aqueous solution, and the negative control group E2 aqueous solution for 2 h.

#### Cold acclimation treatment

A self-designed cold acclimation intelligent cooling system finished the crucian carp's cold acclimation process^15^. The device maintained the circulatory system during the cooling process. Initially, the water temperature was reduced from 20 °C to 10 °C at a cooling rate of 10 °C/h, followed by a further decrease from 10 °C to 5 °C at a rate of 5 °C/h, totaling 2 h. Feeding of crucian carp was disallowed during the cold acclimation phase, and the temperature was monitored in real-time using a high-precision temperature detector. At each temperature point of 20 °C, 10 °C, and 5 °C, six crucian carp were randomly sampled.

#### Determination of indicators

Using a 5-mL disposable syringe, more than 5 mL of blood was drawn from the tail vein after the crucian carp were taken out of the cold acclimation apparatus. For two hours, the blood was refrigerated at 4 °C. Following a 20-min centrifugation at 4000 rpm and 4 °C, the supernatant was removed and kept for later use at − 80 °C in a refrigerator. After the blood was drawn, the crucian carp were cut open on an ice-filled plate. Liquid nitrogen was used to treat the liver and muscle tissues, which were then refrigerated at − 80 °C until needed. Liu et al.^16^ procedure involved selecting a suitable quantity of gill liver tissue at random from two fish and immersing it in a 4% formaldehyde fixative to create paraffin slices.

##### Serum biochemical indicators

The contents of serum glucose, triglyceride (TG), total cholesterol (TC), and urea nitrogen (BUN) were determined by UV spectrophotometry.

##### Metabolic enzyme activity and metabolites

The contents of pyruvate kinase (PK), phosphoenolpyruvate carboxykinase (PEPCK), glycogen, and lactate were measured by UV spectrophotometry, while the contents of glycogen synthase (GCS), fatty acid synthase (FAS), and acetyl-CoA carboxylase (ACC) were determined by enzyme-linked immunosorbent assay.

##### Observation of organizational structure

We observed the gill tissue architectures of the E1, E2, and E3 groups at 5 °C, using the gill tissue of the E1 group at 20 °C as the blank control group. The blank group consists of crucian carp that have undergone neither Vitamin E treatment nor cold acclimation. Using a surgical blade, we carefully cut the gills into slices that were 5–6 µm thick. We then fixed the gill tissues with 4% formaldehyde fixative and performed gradient dehydration, which involved immersing the gills in ethanol solutions with volume fractions of 50%, 70%, 85%, 95%, and absolute ethanol in turn for two hours at a time. The tissues were then fixed in paraffin, sliced, stained with hematoxylin and eosin (HE), and sealed with neutral balsam. Ultimately, an optical microscope was used to see and examine the histological features.

### Statistical analysis

The standard deviation ± mean is used to express all data. Using SPSS 22.0 (Version 22, IBM Corp., Armonk, NY, USA), a statistical analysis of the data was carried out. The analysis employed Duncan's multiple comparison method, which is based on two-way analysis of variance. The minimal threshold of significance was established at *P* < 0.05 and *P* < 0.01 in each instance. The Origin 2019 program was used to plot the outcomes.

## Results

### Effects of vitamin E on serum biochemical indexes under cooling stress

**Figure 1:**
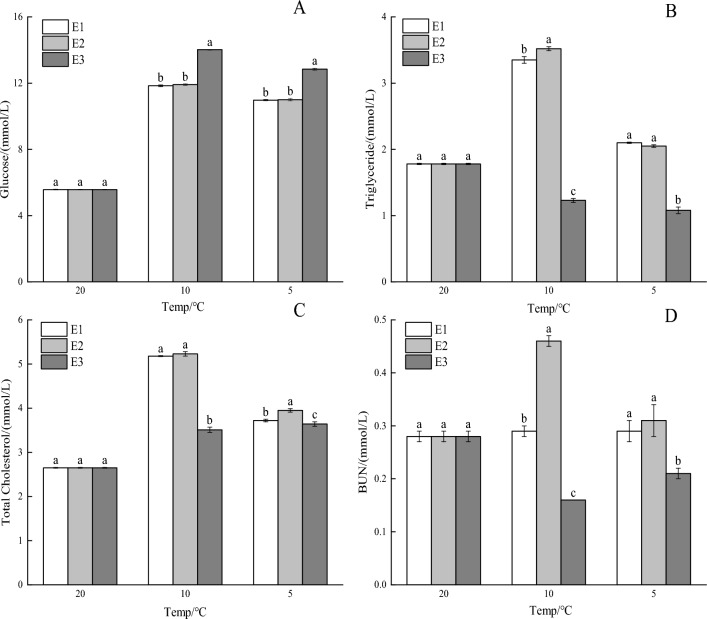
Vitamin E's effects on the serum biochemical indices of (**A**) glucose, (**B**) triglyceride, (**C**) total cholesterol, and (**D**) urea nitrogen (BUN) in crucian carp during cooling stress. Significant differences are shown by different lowercase letters above columns, and no significant differences are indicated by the same lowercase letters.

As the temperature fell, the serum glucose levels in the E1, E2, and E3 treatment groups initially rose and then declined. Notably, the glucose concentration in the E3 group was significantly higher than in the E1 and E2 groups (*P* < 0.01; Fig. 1A). Conversely, during the cooling process, the triglyceride content in the E1 and E2 groups increased initially and then decreased, whereas it continuously decreased in the E3 group. The triglyceride concentration in the E3 group was significantly lower than in the E1 and E2 groups (*P* < 0.01; Fig. 1B). As the temperature dropped, the total cholesterol content in the E1 and E2 treatment groups initially rose and then decreased, whereas in the E3 group, it exhibited an upward trend. Notably, the total cholesterol concentration in the E3 group was significantly lower than in the E1 and E2 groups (*P* < 0.05; Fig. 1C). During the cooling process, the urea nitrogen content in the E1 and E2 groups increased initially and then decreased, whereas it decreased first and then increased in the E3 group. The urea nitrogen concentration in the E3 group was significantly lower than in the E1 and E2 groups (*P* < 0.01; Fig. 1D).

### Effects of vitamin E on glycogen and lactate contents in liver and muscle under cooling stress

**Figure 2:**
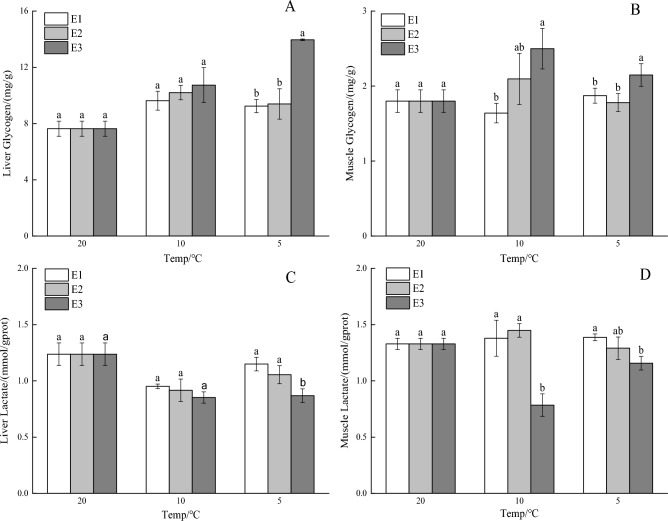
Vitamin E's effects on the amount of glycogen and lactate in the liver and muscle of crucian carp under cooling stress. Significant differences are shown by different lowercase letters above columns, and no significant differences are indicated by the same lowercase letters.

As the temperature decreased, the liver glycogen content in the E1 and E2 groups demonstrated a declining trend, whereas in the E3 group, it initially decreased and then rose (Fig. 2A). Notably, the liver glycogen content in the E3 group was significantly higher than in the E1 and E2 groups (*P* < 0.01). The muscle glycogen content in the E1 group initially decreased and then increased as the temperature fell, whereas it showed a downward trend in the E2 and E3 groups. However, the muscle glycogen content in the E3 group was significantly higher than in the E1 and E2 groups (*P* < 0.05; Fig. 2B). As the temperature fell, the liver lactate content in the E2 and E3 groups initially decreased and then rose, whereas in the E1 group, it exhibited an upward trend (Fig. 2C). Notably, the liver lactate content in the E3 group was significantly lower than in the E1 and E2 groups (*P* < 0.05). As the temperature decreased, the muscle lactate content in the E1 and E2 groups first increased and then decreased, while it decreased initially and then increased in the E3 group. The muscle lactate content in the E3 group was significantly lower than in the E1 and E2 groups (*P* < 0.01; Fig. 2D).

### Effects of vitamin E on enzyme activities related to glucose metabolism under cooling stress

**Figure 3:**
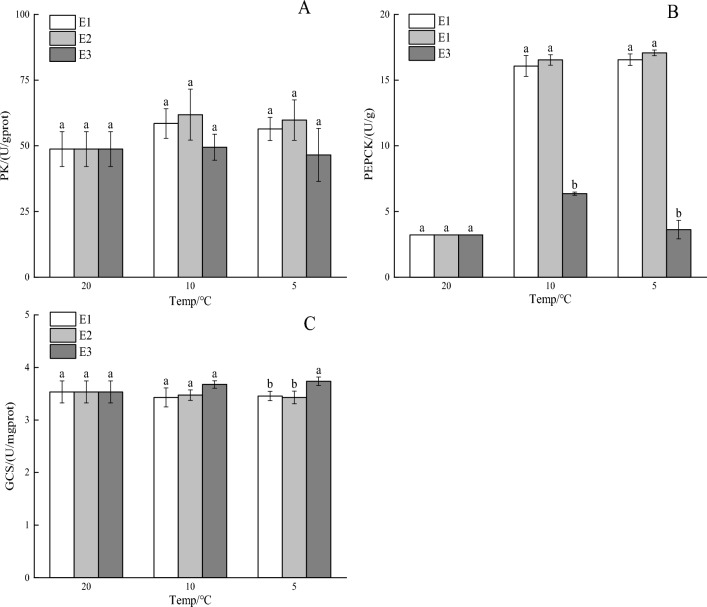
Impact of vitamin E on glucose metabolism-related enzyme activity in crucian carp during cooling stress: (**A**) pyruvate kinase (PK), (**B**) phosphoenolpyruvate carboxykinase (PEPCK), and (**C**) glycogen synthase (GCS) are the three enzymes. Significant differences are shown by different lowercase letters above columns, and no significant differences are indicated by the same lowercase letters.

With the decrease in temperature, the PK activity in the muscle tissue of crucian carp in the E1, E2, and E3 groups initially rose and then fell (Fig. 3A), with no significant differences observed among the groups (*P* > 0.05). As the temperature dropped, the PEPCK activity gradually increased in the E1 and E2 groups, while in the E3 group, it initially rose and then decreased. Notably, the PEPCK activity in the E3 group was significantly lower than that in the E1 and E2 groups (*P* < 0.01; Fig. 3B). Additionally, the GCS activity decreased in the E2 group with decreasing temperature, while in the E1 group, it initially decreased and then increased. In contrast, the GCS activity in the E3 group rose as the temperature fell. Notably, the GCS activity in the E3 group was higher than that in the E1 and E2 groups (*P* < 0.05; Fig. 3C).

### Effects of vitamin E on enzyme activities related to lipid metabolism under cooling stress

**Figure 4:**
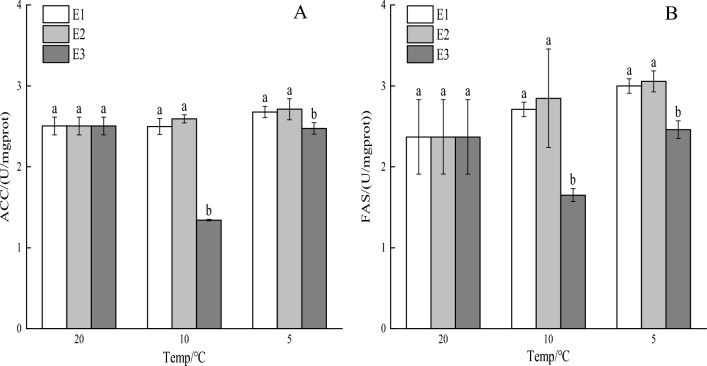
Impact of vitamin E on crucian carp under cooling stress enzymatic activity associated to lipid metabolism: (**A**) acetyl-CoA carboxylase (ACC) and (**B**) fatty acid synthase (FAS). Significant differences are shown by different lowercase letters above columns, and no significant differences are indicated by the same lowercase letters.

As the temperature decreased, the ACC activity in the E1 and E2 groups rose, while it initially dipped and then surged in the E3 group (Fig. 4A). Notably, the ACC activity in the E3 group was significantly lower than that in the E1 and E2 groups (*P* < 0.05). Meanwhile, the FAS activity displayed an upward trend in the E1 and E2 groups with decreasing temperature, whereas it initially decreased and then increased in the E3 group (Fig. 4B). Significantly, the FAS activity in the E3 group was lower than that in the E1 and E2 groups (*P* < 0.05).

### Effects of vitamin E on gill tissue morphology of crucian carp under cooling stress

**Figure 5:**
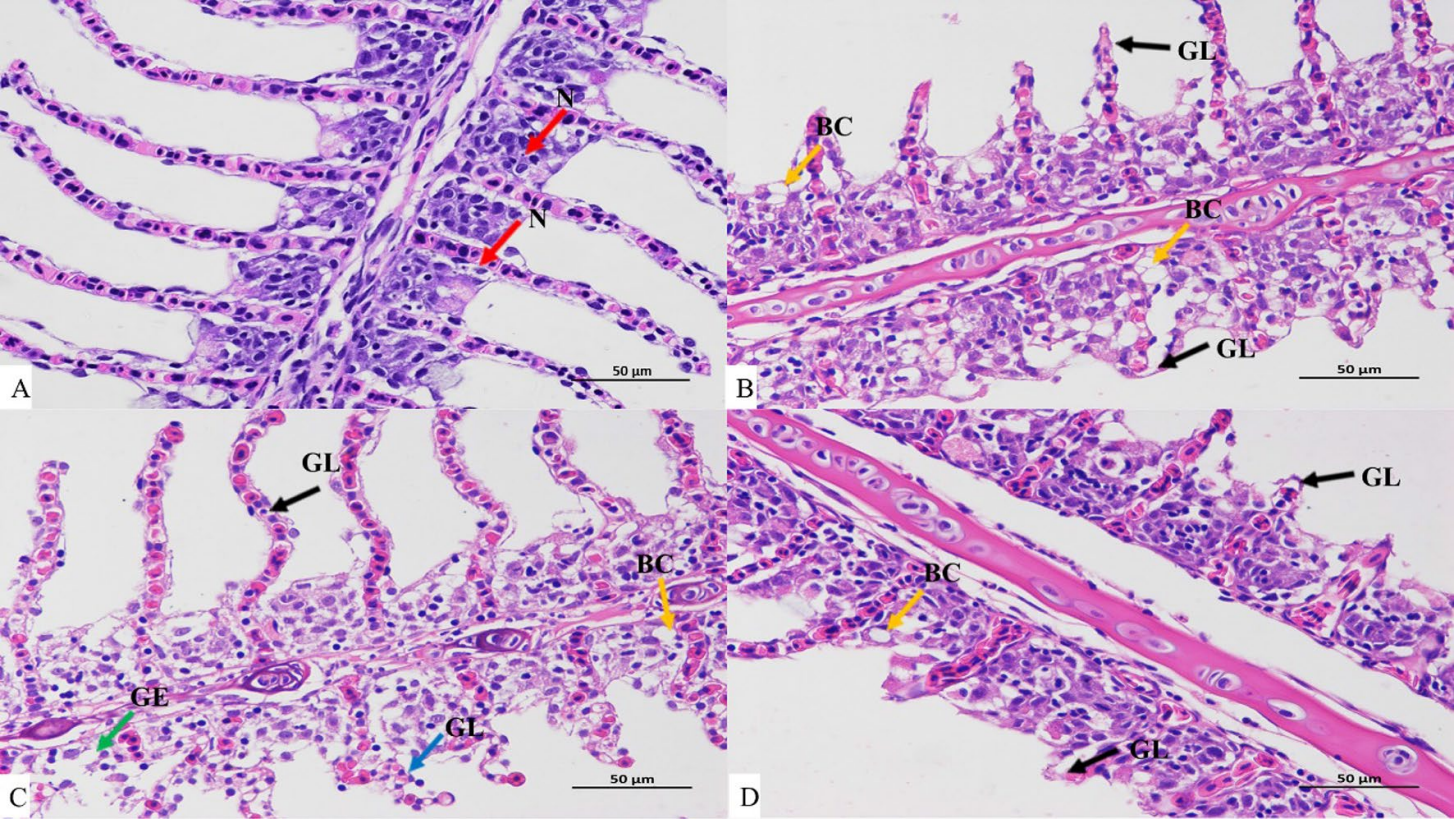
5 °C The impact of vitamin E on Carassius auratus gill tissue structure during cooling stress. (**A**) Blank group, (**B**) E1 group, (**C**) E2 group, (**D**) E3 group, *N* nucleus, *GL* gill lamellae, *GE* gill epithelium, *BC* blood capillary.

The gill filaments in the blank group's gill tissue stretched to both sides under a light microscope, generating a large number of tiny, semi-circular, flat, sac-like lamellae. Gill filament epithelium, which coated the gill filaments, was made up of several layers of flat epithelial cells. Occasionally, necrosis of the epithelium and fragmentation of cell nuclei were seen. Single-layer epithelial cells, capillaries, and columnar supporting cells made constituted the gill lamellae. The flat epithelial cells (Fig. 5A) were attached to the gill filament epithelium. The gill tissue of the E1 treatment group exhibited a small amount of capillary dilatation and decreased lamellae length when compared to the blank group (Fig. 5B). The E2 treatment group's gill tissue displayed a modest amount of capillary dilatation, focal gill filament epithelium separation, irregular lamellae arrangement, and slight lamellae shortening (Fig. 5C). The E3 treatment group's gill tissue had occasionally occurring capillary dilatation and shorter lamellae length (Fig. 5D).

## Discussion

### Effects of vitamin E on glucose metabolism under cooling stress

Glycogen is a material that stores energy. When an organism is under stress, it uses glycogen through the hexose pathway or glycolysis to meet its energy needs^17^. Under lead and cadmium stress, Khanh et al.'s^18^ research revealed that the liver and muscle tissues of climbing perch had much lower glycogen levels than usual. Additionally, Brandao et al^19^ discovered that the large-cap big carp contributes to maintaining energy demand by fending off stress caused by glycogen breakdown during transportation. In our study, the glycogen content in the liver and muscle of crucian carp reduced during the cooling stress treatment, suggesting that carp provided the energy needed for the body to fight cold through glycogen decomposition at low temperature. However, compared to the E1 and E2 groups, the glycogen content in the liver and muscle tissues was much higher for the E3 group. This suggests that the vitamin E-treated carp may be able to somewhat mitigate the cooling stress, which would lessen the body's breakdown of glycogen. The GCS is a key target enzyme for insulin, a rate-limiting enzyme in the liver and muscle, and a crucial player in controlling glucose metabolism and preserving blood glucose homeostasis^20^. The body's GCS activity is lowered in response to external stress, which prevents glycogen production^5,21^. In this investigation, upon cooling stress treatment, the GCS activity in the E1 and E2 groups decreased, and was notably lower than that observed in the E3 group. Correspondingly, the level of glycogen synthesized by the body was also significantly lower in the E1 and E2 groups compared to the E3 group, which aligns with our findings regarding glycogen levels. This suggests that the administration of vitamin E to crucian carp mitigated the inhibitory effect of stress on glycogen synthesis to a certain extent and augmented GCS activity. These findings are comparable to the results reported by Conde-Sieira et al^22^, who studied the anti-stress effects of oral melatonin on rainbow trout.

Since it limits the rate of glycolysis, the enzyme PK is crucial for preserving blood glucose levels^23,24^. According to Wang et al^25^, yellow catfish liver PK activity rose while they were under hypoxic stress, and their body had a significant metabolic reaction. Wang et al^26^ demonstrated that during low-temperature stress, PK activity in the liver of spotted tail perch reached a peak, and glucose metabolism contributed to the body's ability to withstand cold. In our study, muscle's PK activity increased following cooling stress treatment, but there was no discernible difference in PK activity between the E1, E2, and E3 groups. This suggests that, during cooling stress, muscle's glycolysis helped to make up for the energy used to deal with stress; however, vitamin E treatment had no discernible impact on the muscle's glycolysis pathway. In the gluconeogenesis pathway, the enzyme PEPCK is essential. Low-temperature stress primarily causes the body to regulate blood glucose levels through gluconeogenesis, and the rate at which this process occurs is determined by the expression level of this enzyme, according to research on fish stress^27,28^. In a zebrafish study, Vijayan^29^ discovered that starvation stress activated the zebrafish PEPCK gene. In a study on pearl gentian grouper subjected to cold temperatures, there was a considerable increase in liver PEPCK activity and PEPCK expression levels, as well as a rise in blood glucose due to gluconeogenesis^5^. Under cooling stress, PEPCK activity rose in the E1, E2, and E3 groups in our study, but it was significantly lower in the E3 group than in the E1 and E2 groups. This suggests that vitamin E treatment strengthened the crucian carp's resistance to cold and decreased blood glucose consumption.

The blood glucose level rose and crucian carp's gluconeogenesis was boosted during the cooling process. On the other hand, when the temperature dropped, so did the need for blood glucose and its concentration. However, following vitamin E therapy, crucian carp's stress tolerance increased, blood glucose requirements dropped, and blood glucose content was higher than it was without vitamin E treatment—similar to the findings of Dawood et al^30^.

In order to meet its energy needs, the body will break down glycogen while under stress. Lactate is produced by the body's anaerobic metabolism, which occurs when glycogen is broken down^31^. In our research, cooling stress treatment caused a reduction in glycogen content in both liver and muscle tissues, accompanied by an increase in lactic acid levels. Notably, crucian carp treated with vitamin E exhibited significantly lower lactic acid content compared to those without treatment. This observation suggests that vitamin E treatment attenuated the metabolic response of crucian carp to cooling stress.

### Effects of vitamin E on lipid metabolism under cooling stress

An essential transport mechanism for controlling lipid metabolism is blood. TG and TC are the primary constituents of blood lipids. The body's lipid metabolism may be reflected in the levels of serum TG and TC^32–34^. In their study on hybrid yellow catfish, Dagoudo et al.^35^ discovered that acute hypoxic stress treatment raised blood TG and TC levels. Zhang et al.^36^ demonstrated that acute heat stress led to a significant increase in serum TG and TC levels utilizing hybrid yellow catfish as well. According to Chen et al.^37^, grass carp can experience oxidative stress due to oxidized fish oil, and following this stress, there was a considerable increase in the amount of TG and TC in their serum. In our study, cooling stress led to an elevation in serum TG and TC levels, presumably due to the fish's accelerated oxidative metabolism and enhanced intracellular oxygen transport capacity as a means to combat the low-temperature environment. This finding aligns with the results reported by Dagoudo et al.^35^. Notably, the serum TG and TC contents were significantly lower in the E3 group compared to the E1 and E2 groups, suggesting that vitamin E treatment effectively mitigated the stress on the fish's body and moderated its metabolic response.

The end result of the body's metabolism of proteins is the BUN. When fish are under stress, their serum BUN concentration rises and their body accumulates more metabolic waste^38^. Cao et al.^39^ discovered that physiological stress of turbot was generated during transportation, which was demonstrated by a considerable rise in serum urea concentration, by comparing the impacts of water and waterless transportation. It was discovered in a zebrafish investigation that the serum BUN concentration increased with salinity and temperature stress^40^. In our study, the BUN in fish serum rose following cooling stress treatment, yet it was notably lower in the E3 group compared to the E1 and E2 groups. This suggests that vitamin E treatment effectively mitigated the adverse effects of stress and lowered the BUN, echoing the findings reported by Li et al.^41^.

The primary enzymes and variables that control fat deposition are FAS and ACC^42^, and ACC is a crucial regulatory enzyme in the start of fat synthase. Stress from a high-fat diet increased *Macrobrachium rosenbergii* post-larvae's ACC gene expression^43^. An enzyme FAS is a complicated enzyme with multiple functions that is essential for the start of fatty acid synthesis. It was discovered in the *Epinephelus coioides* study that the expression of the FAS gene increased with the degree of oxidative fish oil stress^44^. In our investigation, we found that the crucian carp liver's ACC and FAS activities rose when the fish underwent cooling stress. However, these activities were considerably reduced in the E3 group compared to the E1 and E2 groups, suggesting that vitamin E treatment might lessen lipid metabolism and the fish's body's cooling stress. This agrees with Shao et al.'s^45^ findings.

### Effects of vitamin E on histopathological changes of crucian carp under acute cooling stress

The gill, which serves as a fish's primary respiratory organ, can expel nitrogen, ammonia, and other metabolic waste products in addition to controlling osmotic pressure^46^. Research indicates that ambient temperature is another important environmental component that might impact the osmotic pressure balance and cell membrane permeability of fish, in addition to salinity concentration^47^. Gill tissue cells are impacted by the external environment in two ways: first, through defense mechanisms that result in edema of the respiratory epithelium of gill lamellae, hyperplasia and hypertrophy of gill filament epithelial cells, etc.; second, through direct damage that includes gill epithelial cell necrosis and peeling, etc^48^. According to the study, the *tegillarca granosa* gradually increases the number of cells with a high mitochondrial content as a result of having to expend a lot of energy to re-establish a new balance and maintain the osmotic pressure equilibrium under heavy metal stress. Intense activities and stable osmotic pressure both promote cell respiration, which raises oxygen consumption and necessitates the use of more red blood cells to carry oxygen^49^. The gill lamellae contain a greater number of red blood cells. Stress-induced gill filament vasoconstriction, however, causes an uneven distribution of red blood cells, which accumulates blood cells in specific regions. In this study, the gill tissues of crucian carp treated with vitamin E (E3 treatment group) showed a lighter degree of capillary dilatation, indicating less damage to the gill tissues, in comparison to the gill tissues of crucian carp that were not treated with vitamin E (E1 and E2 treatment groups). Analysis suggests that vitamin E treatment can strengthen the osmotic pressure regulation capacity of fish, subsequently enhancing their immune system and cold resistance in crucian carp during cooling stress, while mitigating gill damage.

## Conclusion

In this study, crucian carp responded to cold stress by elevating the activity of metabolic enzymes to boost metabolism and replenish energy. Vitamin E treatment effectively alleviated the adverse effects of cold stress, leading to a reduction in metabolic enzyme activity. Additionally, this treatment enhanced the fish's cold tolerance and slowed down gill damage. For future research, proteomic analysis is necessary to further explore the mitigating mechanism of vitamin E treatment on crucian carp under cold stress, providing valuable theoretical insights for the application of vitamin E in aquatic environments to alleviate fish stress.

### Supplementary Information

Supplementary Information.

## Data Availability

Additional data are made available in supplementary tables of this manuscript.
