# Supplementary material for: Effect of vitamin E on energy metabolism indicators and gill tissue structure of crucian carp (Carassius auratus) under cooling stress
Source: Sci Rep. 2024 Aug 22;14:19484. doi: 10.1038/s41598-024-66327-z (PMC11341694; doi:10.1038/s41598-024-66327-z)
Supplement: Supplementary file 1 — Supplementary Information. [file 41598_2024_66327_MOESM1_ESM.pdf]

Data Availability Statement:

Additional data are made available in supplementary tables of this manuscript.

Appendix

Fig. 1 Original data:

(1) glucose

	E1			mean value	standard deviation	significance
20	5.58	5.58	5.57	5.577	0.01	a
10	11.81	11.9	11.82	11.843	0.05	b
5	10.98	10.95	11	10.977	0.03	b
	E2			mean value	standard deviation	significance
20	5.58	5.58	5.57	5.577	0.01	a
10	11.91	11.95	11.86	11.907	0.05	b
5	11.08	10.93	10.99	11	0.08	b
	E3			mean value	standard deviation	significance
20	5.58	5.58	5.57	5.577	0.01	a
10	14.08	13.97	14	14.017	0.02	a
5	12.9	12.85	12.78	12.843	0.06	a

(2) triglyceride

	E1			mean value	standard deviation	significance
20	1.79	1.78	1.77	1.78	0.01	a
10	3.41	3.34	3.31	3.35	0.05	b
5	2.11	2.1	2.09	2.1	0.01	a
	E2			mean value	standard deviation	significance
20	1.79	1.78	1.77	1.78	0.01	a
10	3.55	3.51	3.49	3.52	0.03	a
5	2.07	2.04	2.03	2.05	0.02	a
	E3			mean value	standard deviation	significance
20	1.79	1.78	1.77	1.78	0.01	a
10	1.25	1.2	1.23	1.23	0.03	c
5	1.13	1.04	1.08	1.08	0.05	b

(3) total cholesterol

	E1			mean value	standard deviation	significance
20	2.65	2.66	2.64	2.65	0.01	a
10	5.18	5.19	5.18	5.18	0.01	a
5	3.7	3.71	3.76	3.72	0.03	b
	E2			mean value	standard deviation	significance
20	2.65	2.66	2.64	2.65	0.01	a
10	5.29	5.21	5.2	5.23	0.05	a
5	4	3.92	3.93	3.95	0.04	a
	E3			mean value	standard deviation	significance
20	2.65	2.66	2.64	2.65	0.01	a
10	3.45	3.51	3.56	3.51	0.06	b
5	3.59	3.68	3.64	3.64	0.05	c

(4) BUN

	E1			mean value	standard deviation	significance
20	0.29	0.29	0.27	0.28	0.01	a
10	0.3	0.28	0.29	0.29	0.01	b
5	0.27	0.29	0.3	0.29	0.02	a
	E2			mean value	standard deviation	significance
20	0.29	0.29	0.27	0.28	0.01	a
10	0.47	0.45	0.47	0.46	0.01	a
5	0.34	0.31	0.29	0.31	0.03	a
	E3			mean value	standard deviation	significance
20	0.29	0.29	0.27	0.28	0.01	a
10	0.16	0.16	0.16	0.16	0	c
5	0.22	0.2	0.2	0.21	0.01	b

Fig. 2 Original data:

(1) liver glycogen

	E1			mean value	standard deviation	significance
20	11.183	11.493	12.226	11.634	0.54	a
10	9.327	9.147	10.395	9.623	0.67	a
5	8.887	9.074	9.775	9.245	0.47	b
	E2			mean value	standard deviation	significance
20	11.183	11.493	12.226	11.634	0.54	a
10	9.686	10.713	10.207	10.202	0.51	a
5	8.544	10.615	9.025	9.395	1.08	b
	E3			mean value	standard deviation	significance
20	11.183	11.493	12.226	11.634	0.54	a
10	11.479	10.615	10.117	10.737	1.24	a
5	13.999	13.982	13.884	13.955	0.06	a

(2) muscle glycogen

	E1			mean value	standard deviation	significance
20	2.753	2.965	2.679	2.799	0.15	a
10	1.785	1.598	1.533	1.639	0.13	b
5	1.777	1.973	1.867	1.872	0.1	b
	E2			mean value	standard deviation	significance
20	2.753	2.965	2.679	2.799	0.15	a
10	1.785	2.046	2.454	2.095	0.34	ab
5	1.908	1.68	1.753	1.78	0.12	b
	E3			mean value	standard deviation	significance
20	2.753	2.965	2.679	2.799	0.15	a
10	2.796	2.267	2.43	2.498	0.27	a
5	2.315	2.014	2.112	2.147	0.15	a

Fig. 3 Original data:

(1) liver lactate

	E1			mean value	standard deviation	significance
20	0.969	0.916	0.926	0.937	0.03	a
10	0.943	0.974	0.936	0.951	0.02	a
5	1.108	1.221	1.119	1.149	0.06	a
	E2			mean value	standard deviation	significance
20	0.969	0.916	0.926	0.937	0.03	a
10	1.008	0.808	0.931	0.916	0.1	a
5	0.96	1.097	1.108	1.055	0.08	a
	E3			mean value	standard deviation	significance
20	0.969	0.916	0.926	0.937	0.03	a
10	0.799	0.888	0.87	0.852	0.05	a
5	0.823	0.841	0.939	0.868	0.06	b

(2) muscle lactate

	E1			mean value	standard deviation	significance
20	1.275	1.359	1.351	1.328	0.05	a
10	1.329	1.555	1.254	1.379	0.16	a
5	1.366	1.377	1.418	1.387	0.03	a
	E2			mean value	standard deviation	significance
20	1.275	1.359	1.351	1.328	0.05	a
10	1.51	1.453	1.382	1.448	0.06	a
5	1.259	1.21	1.4	1.29	0.1	ab
	E3			mean value	standard deviation	significance
20	1.275	1.359	1.351	1.328	0.05	a
10	0.67	0.833	0.848	0.784	0.1	b
5	1.084	1.209	1.177	1.157	0.06	b

Fig. 4 Original data:

(1) PK

	E1			mean value	standard deviation	significance
20	50.695	41.345	54.15	48.73	6.62	a
10	64.957	55.522	54.973	58.484	5.61	a
5	61.322	55.124	52.81	56.419	4.4	a
	E2			mean value	standard deviation	significance
20	50.695	41.345	54.15	48.73	6.62	a
10	68.192	66.717	50.66	61.856	9.72	a
5	68.06	58.414	52.872	59.782	7.69	a
	E3			mean value	standard deviation	significance
20	50.695	41.345	54.15	48.73	6.62	a
10	54.51	49.262	44.601	49.458	4.96	a
5	58.194	41.037	40.422	46.551	10.09	a

(2) PEPCK

	E1			mean value	standard deviation	significance
20	3.215	3.215	3.215	3.215	0	a
10	16.075	16.86	15.29	16.075	0.79	a
5	16.075	16.935	16.645	16.552	0.44	a
	E2			mean value	standard deviation	significance
20	3.215	3.215	3.215	3.215	0	a
10	16.075	16.795	16.72	16.53	0.4	a
5	16.86	17.07	17.29	17.073	0.22	a
	E3			mean value	standard deviation	significance
20	3.215	3.215	3.215	3.215	0	a
10	6.43	6.215	6.43	6.358	0.12	b
5	3.215	3.215	4.43	3.62	0.7	b

(3) GCS

	E1			mean value	standard deviation	significance
20	3.503	3.34	3.756	3.533	0.21	a
10	3.437	3.604	3.248	3.43	0.18	a
5	3.513	3.498	3.353	3.455	0.09	b
	E2			mean value	standard deviation	significance
20	3.503	3.34	3.756	3.533	0.21	a
10	3.38	3.456	3.583	3.473	0.1	a
5	3.424	3.312	3.55	3.429	0.12	b
	E3			mean value	standard deviation	significance
20	3.503	3.34	3.756	3.533	0.21	a
10	3.755	3.616	3.657	3.676	0.07	a
5	3.771	3.788	3.649	3.736	0.08	a

Fig. 5 Original data:

(1) ACC

	E1			mean value	standard deviation	significance
20	2.454	2.425	2.635	2.505	0.11	a
10	2.6	2.403	2.49	2.498	0.1	a
5	2.729	2.71	2.592	2.677	0.07	a
	E2			mean value	standard deviation	significance
20	2.454	2.425	2.635	2.505	0.11	a
10	2.641	2.601	2.538	2.593	0.05	a
5	2.565	2.78	2.788	2.711	0.13	a
	E3			mean value	standard deviation	significance
20	2.454	2.425	2.635	2.505	0.11	a
10	1.349	1.333	1.342	1.341	0.01	b
5	2.484	2.397	2.542	2.474	0.07	b

(2) FAS

	E1			mean value	standard deviation	significance
20	2.187	2.033	2.887	2.369	0.46	a
10	2.633	2.691	2.804	2.709	0.09	a
5	3.072	2.904	3.018	2.998	0.09	a
	E2			mean value	standard deviation	significance
20	2.187	2.033	2.887	2.369	0.46	a
10	3.225	3.174	2.14	2.846	0.61	a
5	3.14	2.903	3.123	3.055	0.13	a
	E3			mean value	standard deviation	significance
20	2.187	2.033	2.887	2.369	0.46	a
10	1.727	1.57	1.65	1.649	0.08	b
5	2.359	2.45	2.569	2.459	0.11	b
